# Dimethoxytolyl propylresorcinol induces apoptosis and mitophagy in human leukemia cells through the PI3K/AKT pathway

**DOI:** 10.7150/jca.89243

**Published:** 2024-01-01

**Authors:** Jianming Wei, Xiaojuan Zhong, Huiting Peng, Bingqing Cui, Xianlin Yue, Zewei Sun, Jing Shi

**Affiliations:** 1 Department of Pharmacy, Shandong Cancer Hospital and Institute, Shandong First Medical University and Shandong Academy of Medical Sciences, Jinan, Shandong, 250117, China.; 2 School of Pharmacy and Pharmaceutical Science, Shandong First Medical University & Shandong Academy of Medical Sciences, Jinan, Shandong, China.

**Keywords:** dimethoxytolyl propylresorcinol, leukemia, mitophagy, apoptosis, PI3K/AKT

## Abstract

Dimethoxytolyl propylresorcinol (UP302), a natural compound extracted from *Dianella ensifolia,* owing to its tyrosinase inhibitory and strong antioxidant properties, is used in whitening cosmetics. However, the role of UP302 has not been reported in cancer treatment. This study aimed to assess the *in vitro* antitumor activity of UP302 in different tumor cells. It inhibited the growth of certain cancer cell lines and especially in leukemia cells. Therefore, we investigated the antitumor effect of UP302 in leukemia by examining the cell cycle, apoptosis, reactive oxygen species levels (ROS) production, and changes in mitochondrial membrane potential. Our results demonstrated that UP302 inhibited the growth of leukemia cells both *in vivo* and *in vitro* and exerted a proapoptotic effect on MV411 and K562 cells, confirmed by flow cytometry and western blot analysis. Furthermore, UP302 promoted autophagy in MV411 and K562 cells.

Transmission electron microscopy and western blot analysis showed that UP302 induced mitophagy in MV411 and K562 cells. In addition, the autophagy inhibitor chloroquine could enhance UP302-induced apoptosis, suggesting that UP302-mediated autophagy may be protective in MV411 and K562 cells. In conclusion, our study is the first to provide evidence for the anti-leukemia properties of UP302 and the potential clinical use of UP302 combined with autophagy inhibitors as a chemotherapeutic strategy for human leukemia.

## Introduction

UP302 is isolated from the *Dianella ensifolia.* Its main active ingredient is resorcinol, which can effectively inhibit the activity of tyrosinase 1, thereby reducing the production of melanin. It has a strong antioxidant effect and can inhibit free radicals 1-1-diphenylhydroxy-2-hydrazide (DPPH) and lipid oxidation caused by ultraviolet (UV) exposure 2, 3. Therefore, it is used as an ingredient in whitening products.

Leukemia is a unique blood cancer, and despite extensive research into its biology and treatment, targeted therapies for many subtypes are unavailable at present. Generic cytotoxic drugs are the mainstay of treatment, and bone marrow transplantation is the only option 4, 5. Therefore, finding more effective cytostatic drugs is important for the treatment of leukemia.

Degradation of mitochondria through a selective form of autophagy, called mitophagy, is a fundamental mechanism known to be conserved from yeast to humans. It regulates mitochondrial quality and quantity 6, 7. Accumulation of dysfunctional mitochondria is involved in tumorigenesis and mitophagy is important as a tumor-suppressive system 8, 9. Recent studies suggest that inhibiting autophagy may be an efficient approach to improve the efficacy of chemotherapeutic anti-leukemic regimens 10, 11.

The phosphatidylinositol 3-kinase (PI3K), AKT signaling pathway (PI3K/AKT123) is typically dysregulated in disorders related to cell survival and proliferation and is linked with autophagy and apoptosis 12. The pathway can be overactivated in several types of leukemia 13.Many plant-derived molecules have been reported to target this pathway and exert anti-tumor effects.

Therefore, this study, for the first time, aimed to determine if UP302 could inhibit leukemia cell proliferation *in vitro* and *in vivo* and regulate apoptosis and mitophagy. The purpose of this study was to investigate the effect of UP302-induced autophagy on apoptosis and, elucidate the role of the PI3K/AKT pathway in cell death.

## Material and Method

### Materials

UP302 was purchased from Nanjing m&m biotechnology Co. Ltd. The following reagents were used: fetal bovine serum (Procell, China), Dulbecco's Modified Eagle Medium (DMEM), Iscove's Modified Dulbecco's Medium(IMDM), trypsin (Servicebio, China), CCK8 (Sparkjade, China), chloroquine (Sparkjade, China), PI and FITC, Mitochondrial membrane potential assay kit with JC-1 and Reactive Oxygen Species Assay Kit (Beyotime, China), Antibodies against Caspase-3, BAX, BCL-2, LC3B, AKT123, PI3K, P-PI3K, P-AKT, AIF, Tomm20, PINK1, Parkin, Beclin1, and β-actin were purchased from ABclonal(China). Twelve Balb/c female nude mice, aged four weeks, were purchased from Jinan Pengyue Experimental Animal Breeding Co. LTD.

### Cell lines and cultures

Human MV411 and U87MG cells were purchased from Procell. MV411 and K562 cells were grown in IMDM containing 10% fetal bovine serum, 100 U/mL penicillin, and 100 mg/ml streptomycin at 37 °C in an environment with 5% CO_2_. UP302 was dissolved in DMSO and diluted to the final concentrations of 40, 60, 80, 100, and 120 μM. Cells were grown for 24 h or 48 h. Additionally, cells were pretreated with 20 μM chloroquine for 60 min and UP302 treatment was conducted for 16 h to investigate the mechanisms of UP302-induced apoptosis and autophagy. The control group of cells was grown in IMDM.

### Cell viability assay

Cell viability was assessed using the Cell Counting Kit-8 (CCK-8, Sparkjadet, China). Cells were seeded in 96-well plates (1 × 10^4^ cells/well) and incubated without or with UP302 at indicated concentrations (20, 40, 60, 80, 100 and 200μM) for 48 h at 37 °C; five replicated wells were assessed for each group. Subsequently, cells were incubated for an additional 3 h with 20 μL of the CCK-8 reagent at 37 °C. Absorbance values were determined at 450 nm using a microplate reader, and cell viability was calculated.

### Flow cytometry (FCM) detection of ROS, apoptosis, cell cycle, and mitochondrial membrane potential (MMP)

Cells were seeded into a 6-well plate at a density of 4×10^5^ cells/well, and incubated with different concentrations of UP302 (30, 60, 90, and 120 μM) for 16 h, and dye according to the instructions for use. All samples were subject to FCM detection. All data were quantified using FlowJo 10.1.

Cell apoptosis was quantified with double staining for fluorescein isothiocyanate-conjugated Annexin-V and propidium iodide (Biouniquer, China). Treated cells were collected and washed with PBS, resuspended in 100μL 1x binding buffer, and stained with 5μL annexin V-FITC (AV) and 5 μL propidium iodide (PI) for 20 min at 37 ℃ in the dark. Subsequently, 400μL of 1x binding buffer was added.

ROS levels were determined using 2',7'-dichloro-dihydro fluorescein diacetate (H2-DCFDA, Beyotime Biotechnology, China). Propidium iodide was used to assess the cell cycle. MMP was measured using a JC-1 probe. Assays were performed in triplicate and repeated thrice independently.

### Transmission electron microscopy

After treatment with 50μM UP302 for 16 h, cells were collected and washed twice with PBS, and fixed with 2.5% glutaraldehyde for 120 min at room temperature. Subsequently, the cells were fixed with 1% osmium tetroxide for 30 min. After gradient dehydration with ethanol solutions from 50% to 100% in a 10% graded series, cells were embedded in the Epon 812 resin. The blocks were cut into ultrathin sections using a microtome. Sections were stained with saturated uranyl acetate and lead citrate. The autophagosomes were observed using a transmission electron microscope (HITACHI, HT7800).

### Western blot analysis

After treating MV411 and K562 cells with 0, 30, 60, and 90 μM UP302 for 16 h, the collected samples were washed with cold PBS. Cells were lysed in the lysis buffer. After incubation for 30 min at 4 °C, samples were centrifuged at 15,000 rpm for 20 min at 4 °C, and the supernatants were collected for the determination of protein concentration using the BCA protein assay kit (Vazyme, China). The cell extracts were separated on 12% SDS-PAGE gels, transferred onto the PVDF membrane, and blocked in 5% skim milk powder in TBST. Western blot analyses were performed using primary antibody solutions following the manufacturer's instructions. The blot was washed thrice with TBST, and incubated with secondary antibodies (horseradish peroxidase-conjugated goat anti-mouse or goat anti-rabbit antibodies) for 1 h at room temperature. The protein bands were detected using the ECL chemiluminescence system.

### Tumor xenograft model

Twelve-4-week-old female Balb/c nude mice were randomly divided into the control and treatment groups. All of the mice received a subcutaneous injection of 8×10^6 cells (100μl cell suspension) in the right back. The tumor grows to about 100 mm^3^ and treatment is initiated. Mice in the treatment group were intraperitoneally injected with UP302, 10 mg/kg, every two days for two weeks according to our preliminary experimental results, while the control group received an intraperitoneal injection of PBS similarly.

Tumor volume and mouse weight were measured every other day for two weeks. Nude mice were sacrificed and tumor tissues were excised. The volume of the tumor was calculated according to the formula: volume (mm^3^) = ½ ×(length×width ×square). All experimental protocols complied with the guidelines of the Experimental Animal Ethics Committee, Cancer Hospital Affiliated to Shandong First Medical University (Ethics Approval No.: SDTHEC2021007014).

### HE staining

The slides were deparaffinized, stained with hematoxylin for 2 min, washed with distilled water, treated with 0.1% hydrochloric acid and LiCO3 separately, washed again with tap water for 15 min, stained with eosin for 1 min, and dehydrated with gradient alcohol. Xylene was used to turn sections transparent and neutral gum was used for sealing. Research grade orthographic microscope was used to observe and examine the images of sample slides.

### Statistical analysis

Data are presented as the mean ± standard deviation. Differences between the two groups were investigated using the Student's t-test. P < 0.05 indicated a statistically significant difference.

## Results

### Cytotoxic activity of UP302

**Figure 1:**
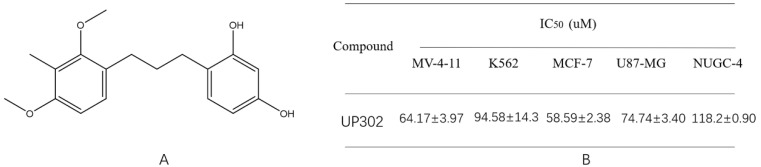
(A): Structure of UP302. (B): cytotoxic activity of UP302 after 48 h of treatment (Units without additional explanation are μmol/L, for short is μM).

To investigate the effect of UP302 (Fig. 1**A**) on cancer cell growth, we selected five tumor cell types: MV411, K562, NUGC-4, U87, and MCF-7, and treated them with different concentrations of UP302 for 48 h, cell viability was evaluated using a CCK-8 assay. The IC50 values were calculated from dose-response curves using GraphPad Prism 5 (Fig. 1**B**), UP302 exhibited antiproliferative activity in leukemia cells MV411 and K562 and three solid tumor cell types. This is the first proof that UP302 has a wide range of anti-tumor activities *in vitro*.

### UP302 induces the production of reactive oxygen species in leukemia cells MV411 and K562, thereby reducing MMP

**Figure 2:**
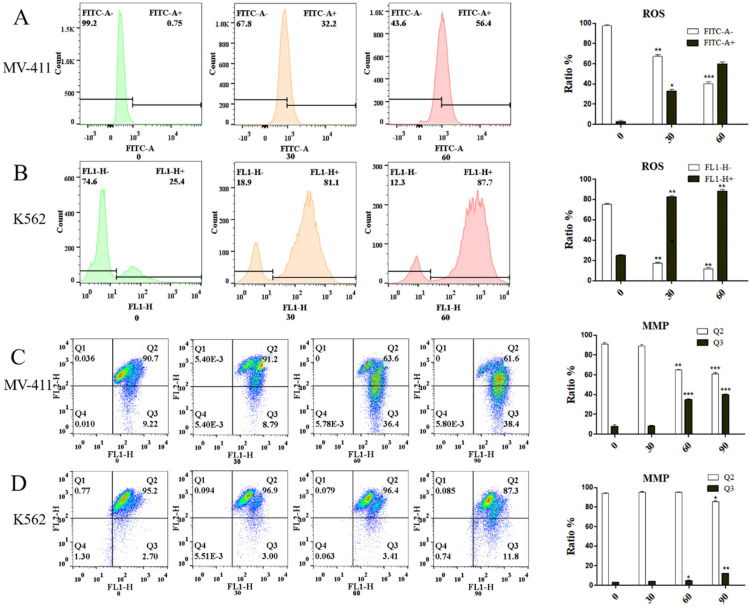
Using the DCFH-DA probe, we demonstrated the effect of UP302 on ROS production in MV411 and K562 cells by flow cytometry. The effect of UP302 on the mitochondrial membrane potential of MV41 and K562 cells was demonstrated by flow cytometry using the JC-1 probe. A: UP302 induces ROS production in MV411 cells. B: UP302 induces ROS production in K562 cells. C: Mitochondrial membrane potential depolarization in MV411 cells treated with UP302. D: Mitochondrial membrane potential depolarization in K562 cells treated with UP302. All data are representative of three independent experiments. ^*^ p < 0.05, ** p < 0.01, *** p < 0.001.

FCM detection showed that leukemic cells, UP302 treatment induced the production of ROS in MV411 and K562 for a short period of time in a dose-dependent manner (Fig. 2**A**, Fig. 2**B**). Too much reactive oxygen in cells causes uncontrolled oxidation and redox stress, which can lead to severe cellular damage and untimely cell death. Redox homeostasis is a key factor for the proper functioning of mitochondria, cells, and organisms 14, therefore, the generation of ROS is also an important aspect of the anticancer effect of UP302. Similarly, MMP depolarization, closely related to ROS, was also observed after UP302 treatment (Fig. 2**C**, Fig. 2**D**). This shows that UP302 is not only a super antioxidant but also induces the production of ROS in tumor cells, thus affecting the MMP, which eventually leads to tumor cell death. However, mechanistic details remain to be explored.

### UP302 induces G2/M phase cell cycle arrest in MV411 and K562 cells

**Figure 3:**
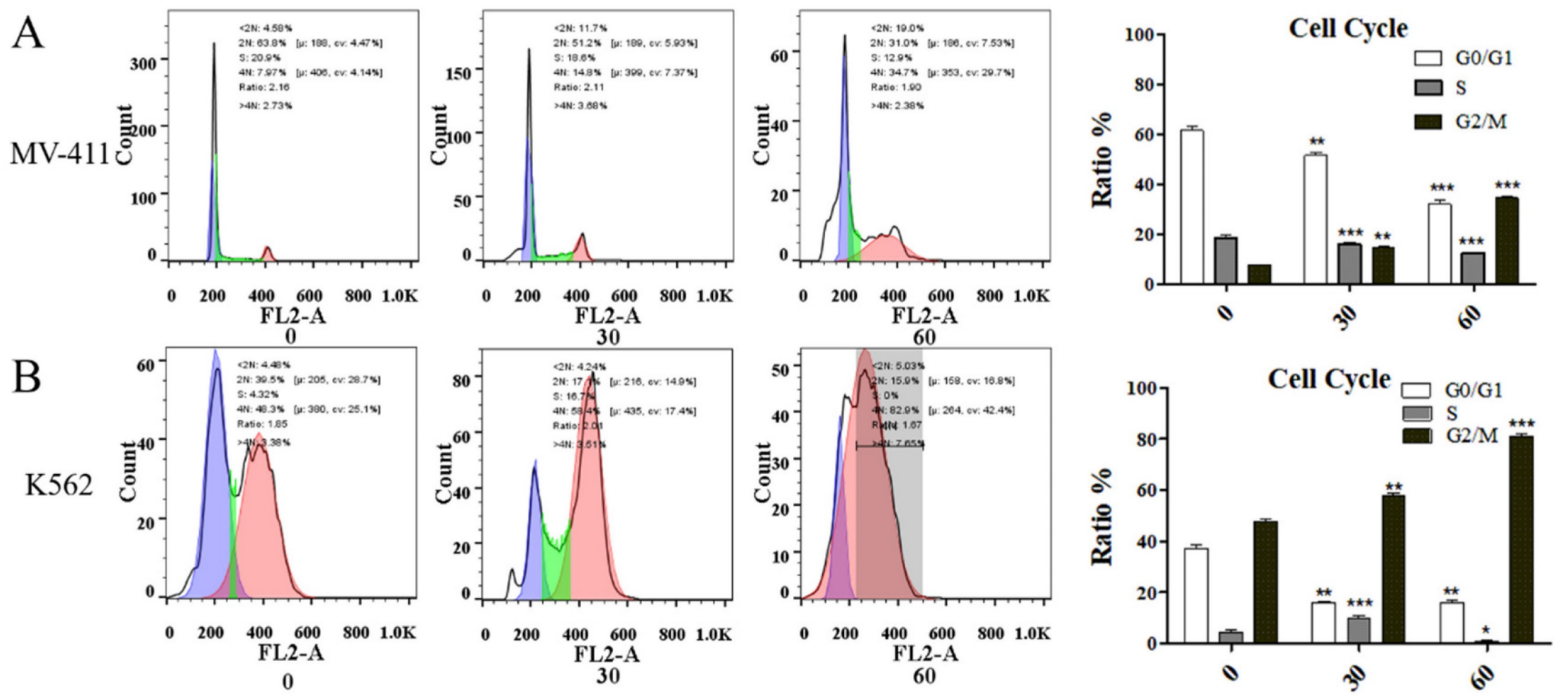
** (**A): The effect of UP302 treatment for 16 h on the cell cycle of MV411, showing arrested G2/M phase. B: The effect of UP302 treatment for 16 h on the cell cycle of K562, showing arrested G2/M phase.

UP302 caused cell cycle arrest in MV411 and K562 cells (Fig. 3**A**, Fig. 3**B**). In a short period, compared with the control group, the treatment blocked the G2/M phase cycle. Cyclin B1 protein is associated with regulatory transitions in the G2/M phase. Western blot results showed that the expression of Cyclin B1 was significantly reduced in UP302-treated cells, indicating that UP302 could effectively block the G2/M phase in MV411 and K562 cells. Despite the highly proliferative nature of leukemia cells, UP302 also can be significantly affected by short-term drug treatment.

**Figure 4:**
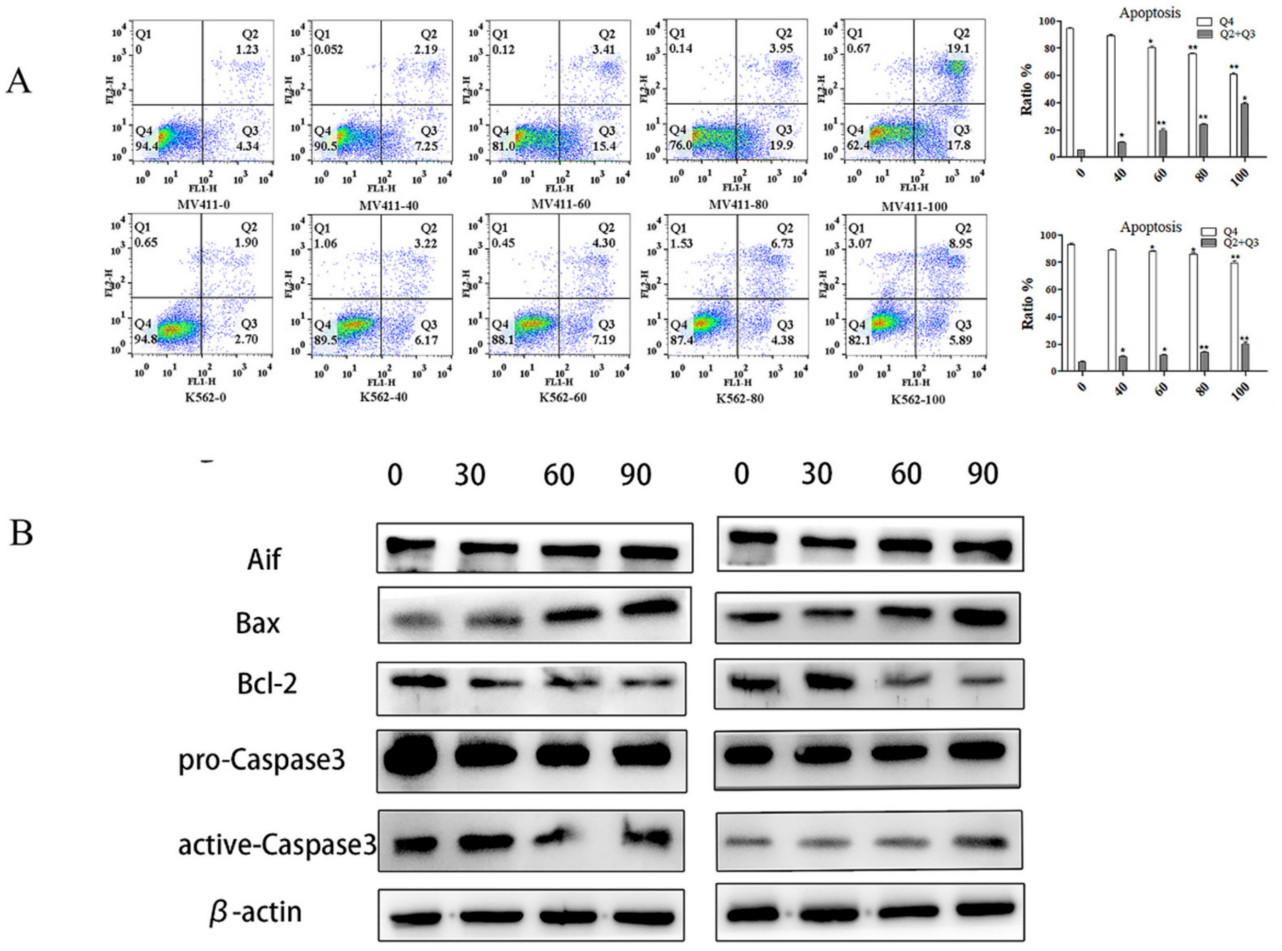
UP302 induced apoptosis in leukemia cells. (A): Apoptosis assays in MV411 and K562 cells treated with UP302 (0-100μM) for 16 h by flow cytometry detection. (B): The protein expression of Aif, Bax-9, Bcl-2, pro-caspase 3, and active-caspase 3 in MV411 and K562 cells was detected by western blotting after treatment with UP302 (0, 30, 60and 90μM) for 5 h.

With the increase in UP302 concentration, the apoptosis rates increased gradually as shown by FCM results. Compared with the control group, both in MV411 and K562, the treatment group (100μM) had significantly increased apoptosis (Fig. 4**A**).

Western blot showed the apoptosis‑inducing factor (AIF), Bax, and active-caspase 3 were overexpressed after 100μM UP302 treatment, while relative protein levels of Bcl-2 were down-regulated in the 100μM UP302 treatment group compared with the control group (Fig. 4**B**).

These results indicated that UP302 could induce apoptosis in leukemia cells, MV411 and K562.

### UP302 induces mitophagy in MV411 and K562 cells

Transmission electron microscopy is used to observe autophagy. We used this method to detect mitochondria and autophagosomes.

**Figure 5:**
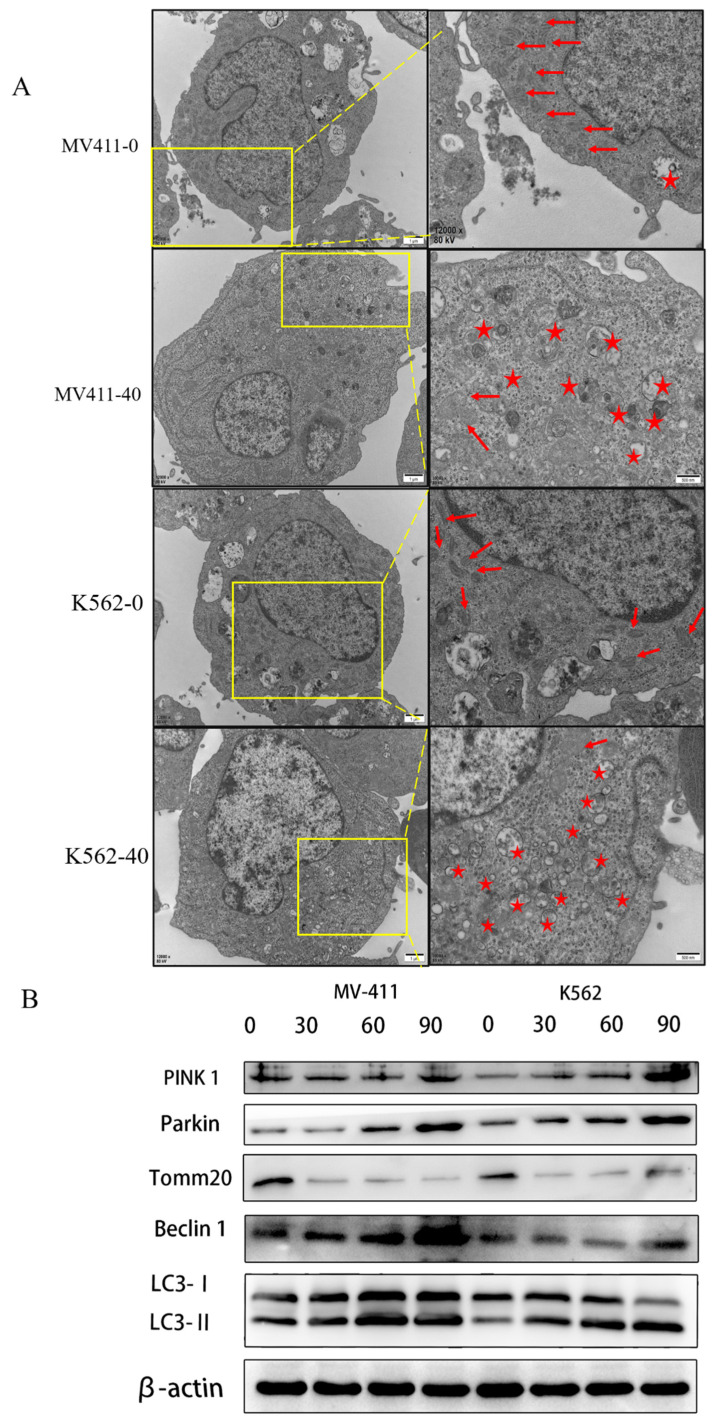
UP302 induced mitophagy in MV411 and K562. (A): After 16 h of treatment with UP302 (50μM), cell ultrastructure was observed by transmission electron microscopy at a magnification of 12000× scale bar, red stars represent autophagosomes, and red arrows represent mitochondria. (B): The protein expression of Pink 1, Parkin, Tomm20, Beclin1, LC3-I, and LC3-II in MV411 and K562 cells was detected by western blot analysis after treatment with UP302 (0, 30, 60, and 90μM) for 5 h.

The results showed several mitochondria and few autophagosomes in the untreated group in both MV411 and K562 cells, while several autophagosomes were observed in the treated group accompanied by the loss of mitochondria. We confirmed that UP302 induced autophagy in MV411 and K562 cells (Fig. 5**A**).

LC3-Ⅱ levels correspond to the level of autophagy; thus, we measured autophagy by quantifying LC3-Ⅱ expression. Both in the MV411 and K562 cells, the levels of LC3-II were dramatically increased in the treatment group compared with the control group, as shown by western blot analysis.

Previous studies suggest that cancer mitophagy is primarily controlled by the PINK1/Parkin pathway (Yan et al. 2018; Zhao et al. 2018). Accordingly, we checked the involvement of the PINK1/Parkin pathway in UP302-related mitophagy. Western blotting assays demonstrated that the levels of both PINK1 and Parkin expression were upregulated (Fig. 5**B**).

The above data demonstrated that UP302 activates mitophagy in MV411 and K562 cells through the PINK1/Parkin pathway.

### UP302 inhibits the activation of the PI3K/AKTsignaling pathway

**Figure 6:**
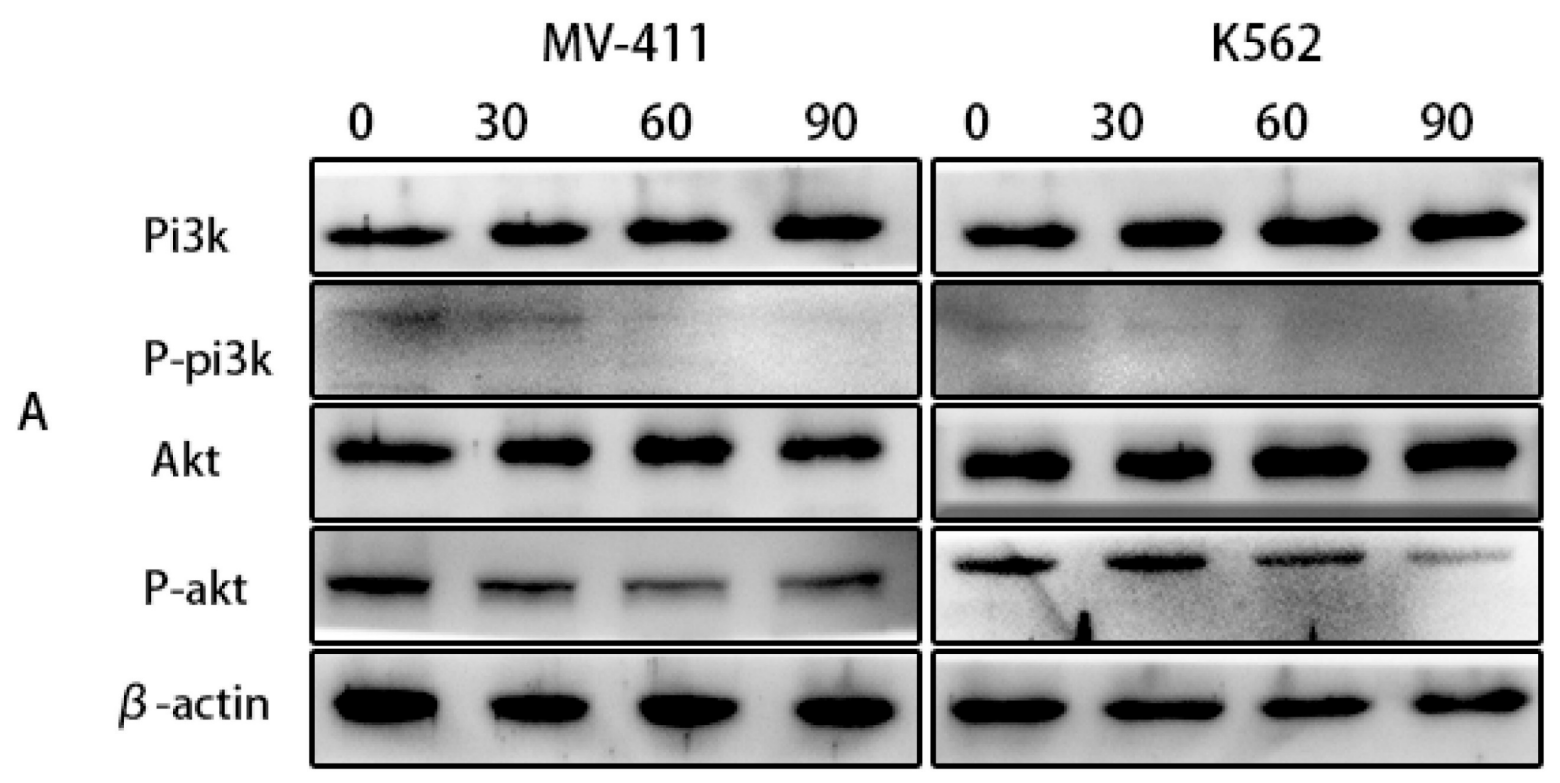
(A): The protein expression of PI3K, P-PI3K, AKT, and P-AKT in MV411 and K562 cells was detected by western blotting after treatment with UP302 (0, 30, 60, and 90 μM) for 5 h.

The phosphatidylinositol 3-kinase (PI3K)/AKT signaling pathway (PI3K/AKT) is frequently dysregulated in disorders of cell growth and survival, including a number of pediatric hematologic malignancies. Therefore, inhibition of this signaling pathway is also a potential therapeutic option for leukemia. According to our observations in the western blotting, UP302 significantly inhibited the phosphorylation of PI3K/AKT (Fig. 6**A**). Therefore, we inferred that UP302 could inhibit this signaling pathway.

### Inhibition of autophagy enhances UP302-induced apoptosis in MV411 and K562 cells

**Figure 7:**
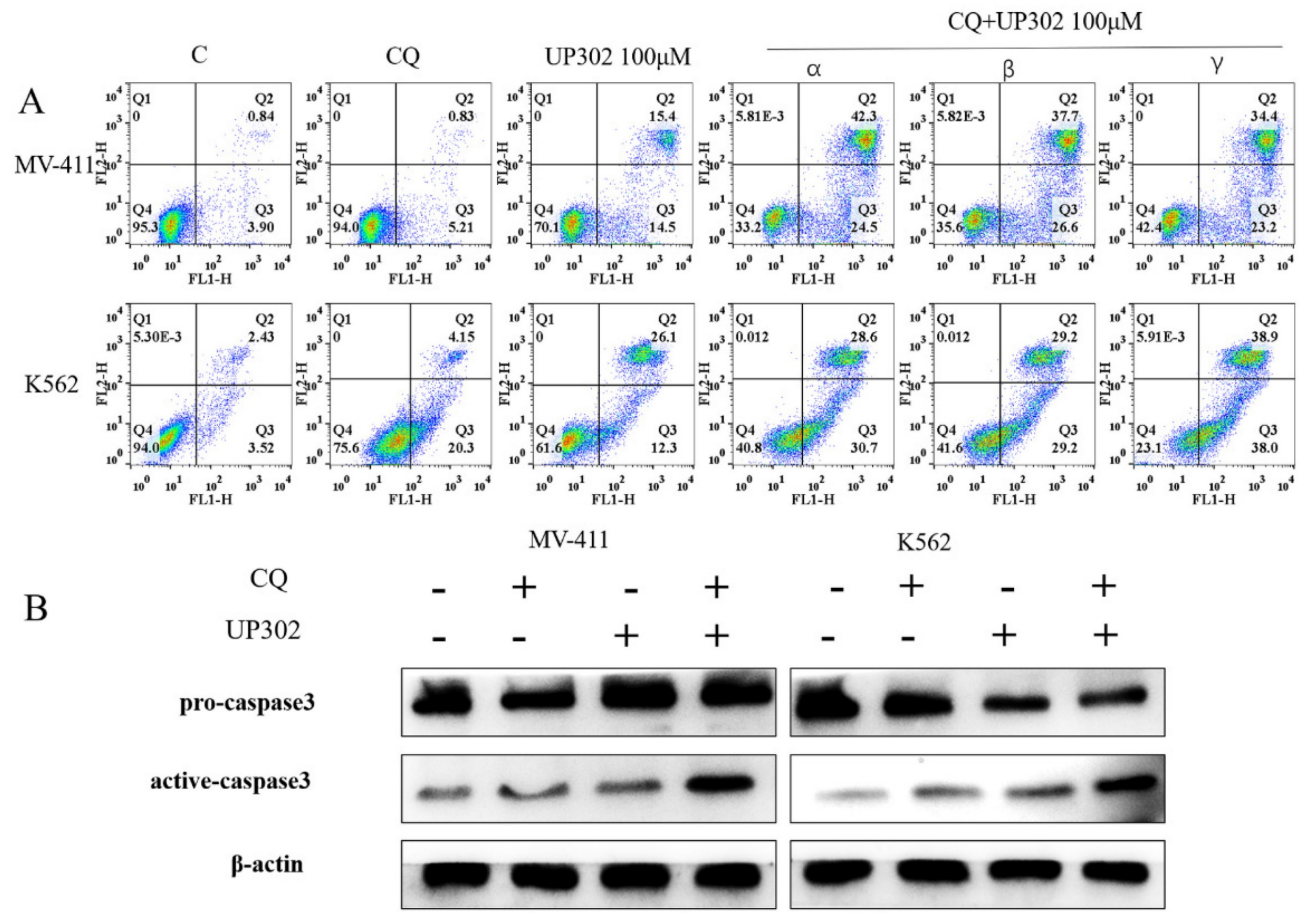
(A): α: Chloroquine was added for one hour, followed by UP302 treatment; β: UP302 was added for one hour followed by chloroquine treatment; γ: Chloroquine and UP302 were added. After adding chloroquine or UP302, or combined treatment in MV411 and K562 cells for 16 h, apoptosis was detected by flow cytometry. (B): After adding chloroquine or UP302, or combined treatment in MV411 and K562 cells for 16 h, western blotting was used to detect proteins related to apoptosis.

To confirm the relationship between the autophagy and apoptosis induced by UP302, cells were treated with 100μM UP302 for 48 h with or without pretreatment with 20μM chloroquine for 60 min. FCM showed that the number of apoptotic cells in the UP302 treatment group was significantly increased after autophagy inhibition (Fig. 7**A**). Western blotting also showed apoptosis of tumor cells in the combination group (Fig. 7**B**).

This result suggests that UP302-induced autophagy is protective in leukemia cells, MV411 and K562.

### UP302 inhibits tumor formation in nude mice and short-term safety evaluation

**Figure 8:**
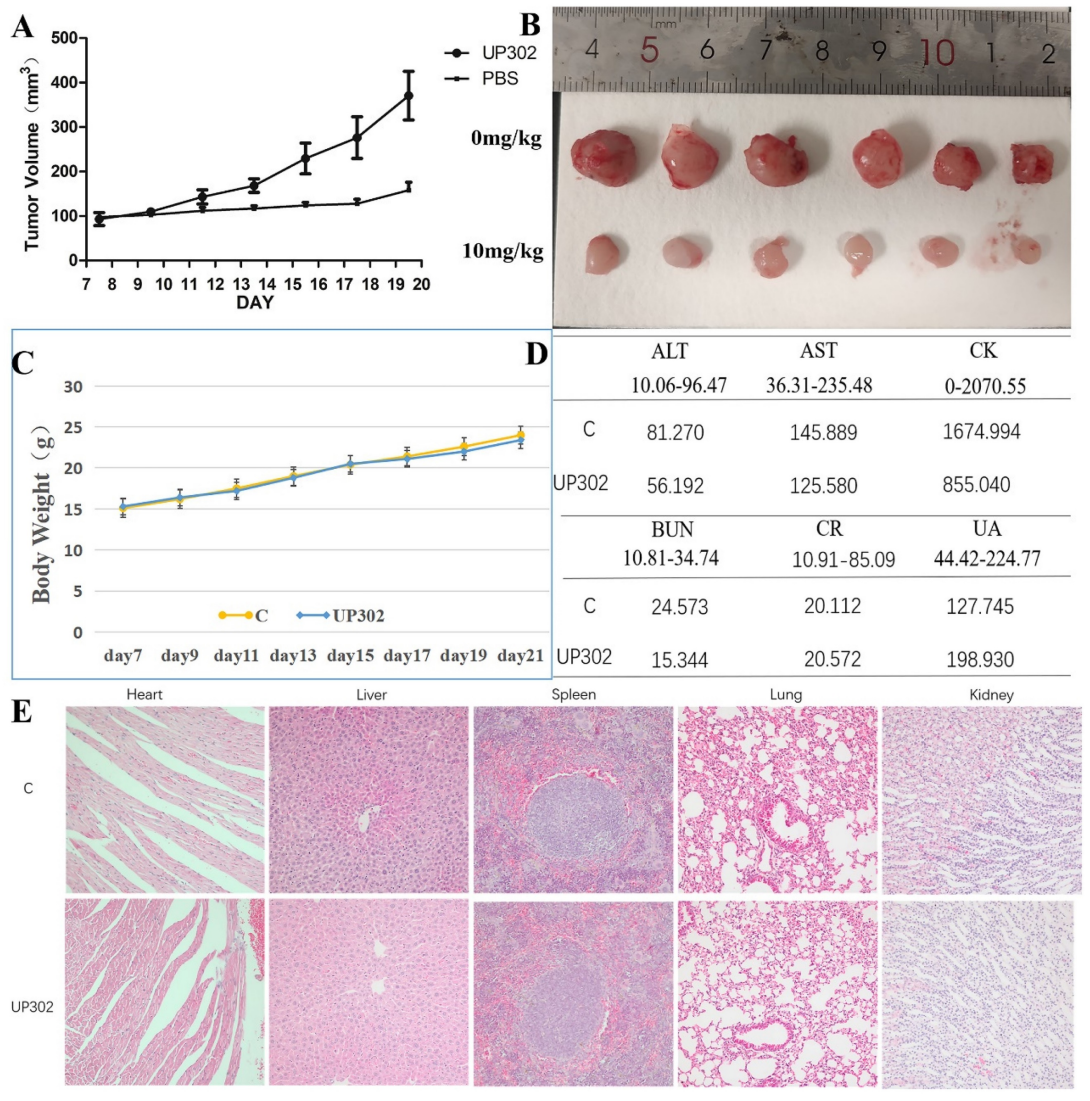
The tumor growth was significantly suppressed by the UP302 and short-term safety evaluation. (A): Mice bearing MV411 tumor xenografts were treated with PBS or UP302, as indicated the tumor volumes were measured every two days. (B): The weights and photographs of isolated tumors. (C): Weight curve. (D): The function of the liver (ALT and AST), kidney (Crea, Urea, and UA), and heart (CK) were evaluated using biochemical blood markers. (E): H&E staining of major organs obtained from different groups.

The antitumor effect of UP302 *in vivo* was evaluated in the subcutaneous tumor model of MV411 tumor grafts. Mice bearing MV411 tumor xenografts were randomly divided into two groups (n=6) and respectively treated with (PBS) or UP302, when the tumor volumes reached approximately 100 mm^3^. As shown in Figures 8**A** and **B**, in the treatment group, the tumor volume reduced significantly, a finding that further confirmed that UP302 was effective in inhibiting tumor cell growth. To evaluate the short-term safety of UP302 *in vivo*, the body weights of mice were recorded every two days. As shown in Figure 8**C**, there was no significant difference in body weight among the groups. After treatment, blood biochemical indicators for functions of the liver (ALT and AST), kidney (CREA, urea, and UA), and heart (CK) of mice in each group were analyzed, and all these biochemical indicators were within the normal reference range (Fig. 8**D**). H&E staining of the major organs (heart, liver, spleen, lung, and kidney) from all treated mice showed no prominent abnormal structures compared to the PBS group (Fig. 8**F**). These results indicated that UP302 exhibited good biosafety during the short-term treatment period at this dose.

## Discussion

UP302 is a natural small molecule extracted from *Dianella ensifolia* with tyrosine kinase inhibitory and antioxidant properties 1, 2. Therefore, it has been used as a whitening ingredient in cosmetics 2. Recently, however, new natural molecules were extracted from *Dianella ensifolia*, some of which showed excellent anti-tumor cell proliferation activity *in vitro* 15, 16. Therefore, we speculate whether UP302 also has a similar function. No study has reported the antitumor effect of UP302.

For the first time, we found that UP302 had a dose-dependent effect on the proliferation of MV411 and K562 leukemia cells, and significantly inhibited the progression of subcutaneous transplantation *in vivo*. Furthermore, as hypothesized, we found that UP302 promoted apoptosis in MV411 and K562 cells. Furthermore, our study showed that the mitochondria of leukemic cells were damaged after UP302 treatment, resulting in a decrease in MMP. Western blotting showed that UP302 treatment increased AIF, active Caspase 3, Bax, Pink 1, Parkin, and LC3-Ⅱ and inhibited the phosphorylation of PI3K and AKT proteins. These results indicate that UP302 induces the apoptosis and autophagy of leukemia cells mainly through the mitochondrial pathway.

Degradation of mitochondria occurs through a selective form of autophagy, named mitophagy. Some targeted drugs, such as histone deacetylases (HDACs), induce autophagy in leukemia but the resulting autophagy promotes survival. Multiple studies have shown that autophagy may be a pro-survival mechanism in hematopoietic diseases, and autophagy activation can promote resistance to standard chemotherapy. Therefore, targeting autophagy may be a promising strategy for combination therapy.

In this study, we confirmed that UP302 induces mitophagy in MV411 and K562 leukemia cells by transmission electron microscopy and detection of autophagy-related proteins. To determine the role of UP302-induced autophagy in leukemia treatment, further functional analysis showed that CQ-mediated mitophagy inhibition significantly increased UP302-induced apoptosis. UP302 had a protective effect on autophagy and the combination of autophagy inhibitors promoted cell death.

In conclusion, for the first time, our study comprehensively demonstrated that UP302, a natural compound extracted from *Dianella ensifolia*, has antitumor characteristics. UP302 inhibited the proliferation of leukemia cells *in vitro* and *in vivo* and induced apoptosis and mitophagy. UP302 inhibited the phosphorylation of the PI3K/AKT signaling pathway. UP302-mediated autophagy is a mechanism that protects leukemia cells from external attacks. The above results may provide an experimental basis for the clinical application of UP302 combined with autophagy inhibitors as a chemotherapeutic strategy against human leukemia.
